# Combined use of *Bacillus* strains and Miscanthus for accelerating biodegradation of poly(lactic acid) and poly(ethylene terephthalate)

**DOI:** 10.7717/peerj.10957

**Published:** 2021-03-30

**Authors:** Grażyna B. Dąbrowska, Katarzyna Janczak, Agnieszka Richert

**Affiliations:** 1 Department of Genetics, Faculty of Biological and Veterinary Science, Nicolaus Copernicus University, Toruń, Kuyavian-Pomeranian, Poland; 2 Research Network Łukasiewicz, Institute for Engineering of Polymer Materials and Dyes, Research Network Łukasiewicz, Toruń, Kuyavian-Pomeranian, Poland

**Keywords:** *Bacillus* sp., Biodegradation, Biofilm, Metabolic Activity, PLA, PET

## Abstract

**Background:**

The aim of this study was to verify whether the presence of *Bacillus* strains and of miscanthus influence biodegradation and formed of biofilm of poly(lactic acid) (PLA) and poly(ethylene terephthalate) (PET).

**Methods:**

The experiment conducted in compost soil showed that strains *Bacillus subtilis* and *Bacillus cereus* isolated from heavy metal contaminated environment have biochemical activity and accelerate biodegradation of both plastic materials.

**Results:**

For PLA film it was found that the carbonyl index dropped by over 15% in the presence of *B. subtilis*, while the film tensile strength decreased by 35% and the oxygen to carbon O/C ratio was higher by 3% in the presence of *B. cereus*, and the presence of miscanthus resulted in a loss of weight. For PET film, a decrease in the carbonyl index by 16% was observed following inoculation with *B. cereus.* The metabolic activity of this strain contributed to the reduction of the film’s tensile strength by 17% and to the increase in the permeability to O_2_ and CO_2_. The most intense degradation of PET film was observed in the presence of bacteria and plants. *B. subtilis* strain combined with miscanthus plantings may be a promising method for accelerating PLA and PET degradation in compost soil.

## Introduction

Each year, 140 million tones of petroleum-based plastics are used all over the world, and the majority of them ends up as waste in the environment (Nampoothiri, Nair & John, 2010). To minimise quantities of pollutants, increasing emphasis is being put on the use of biodegradable materials that easily decompose after being deposited in the environment (Jain et al., 2015). Traditional methods of cleaning pollution are confined to physical and chemical processes. These methods are expensive, time-consuming, and less effective (Ghanem, Al-Garni & Al-Zahrani, 2016).

One of the most common biodegradable plastics is poly(lactic acid) (PLA) of the for food packaging applications. The material is used for hygienic purposes and protection from food pathogens (Prapruddivongs & Sombatsompop, 2013; Richert et al., 2019). It is estimated that about 150.000 tones of PLA are manufactured per annum (Ahmed et al., 2018). PLA is frequently considered as a biodegradable equivalent of poly(ethylene terephthalate) (PET) due to its similar tensile strength, modulus of elasticity, impact resistance, and barrier properties (Malinowski et al., 2016).

One of the polymers synthesised from petroleum is PET, belonging to the aromatic polyesters, and its basic components are terephthalate acid and ethylene glycols. Due to its properties and low price, PET is widely used, for example, in production of packaging, such as bottles or containers (Eubeler, Bernhard & Knepper, 2010). Each year, 13 million tons of this plastic material are used in manufacturing (George & Kurian, 2014). The degradation time of PET in the environment is estimated at 16 to 48 years (Nowak et al., 2008). Most reports on the biodegradation of PET relate to process of hydrolysis, alcoholysis, glycolysis, ammonolysis and aminolysis (Teotia, Tarannum & Kumar, 2017). However, under the influence of temperature, humidity and/or in a presence of materials not hazardous to the environment it should ultimately decompose to carbon dioxide, methane, water and biomass (Gutowska et al., 2014). Although global production is dominated by synthetic materials, nevertheless environmental and economic aspects associated with decreasing petroleum resources result in their replacement with biodegradable materials (Kumar & Maiti, 2016).

In the natural environment, polymer materials are degraded both by biotic factors (microorganisms, bacteria and fungi in particular, and plants) and abiotic factors (solar radiation, atmospheric contamination, heat, and water) (Muenmee, Chiemchaisri & Chiemchaisri, 2015; Nowak et al., 2008). The course of polymer degradation primarily depends on the chemical composition, molecular mass, wettability, crystallinity, concentrations and types of additives, and properties of the surface layer, among others (Vieira, Guedes & Tita, 2013; Zenkiewicz et al., 2013). Bacteria using plastics as a source of carbon and energy are additionally very opportunistic and can adapt to various environments (Das & Kumar, 2015). Development of biodegradation changes, on the other hand, is associated with microorganisms’ ability to produce hydrolytic enzymes such as: esterases, proteases and lipases (Novotný et al., 2015).

There are certain known microorganisms for which it has been demonstrated that they are able to accelerate degradation changes in plastic in PLA both in laboratory conditions (Janczak et al., 2014a; Janczak et al., 2014b) and in soil (Santonja-Blasco et al., 2010; Weng et al., 2013; Lv et al., 2017). Research has been initiated to isolate microorganisms accelerating biodegradation of PET. Yoshida et al. (2016) isolated bacteria *Ideonella sakaiensis* 201-F6, organisms able to produce PET hydrolase (PETase), from an environment contaminated with PET bottle waste. Auta, Emenike & Fauziah (2017) isolated eight bacteria strains from a mangrove ecosystem on seacoasts of Peninsular Malaysia, of which two, *B. cereus* and *Bacillus gottheilii*, demonstrated a potential for biodegradation of materials including PET microplastic (Auta, Emenike & Fauziah, 2017).

Spore-forming Gram-positive bacteria from the *Bacillus* genus are resistant to adverse environmental conditions, including chemicals such as those released by polymer synthetic materials, radiation, or drying (Kunst et al., 1997; Heydarnezhad et al., 2018). Use of *Bacillus* strains for biodegradation of plastics is supported by their high rate of cell divisions, and secretion of many active proteins outside cell in quantities up to 25 g/L, as well as the lack of toxicity and pathogenicity of these strains (Saxena & Stotzky, 2001). The pathogenic potential of *B*. *cereus* is quite variable, ranging from strains used as plant growth promoter and biopesticides to strains causing fatal diseases. *B*. *cereus* is well known as important foodborne pathogen, which can cause two different types of gastrointestinal diseases (Messelhäußer, Ehling-Schulz, 2018).

*Bacillus* sp. strains used in research belong to *Plant Growth Promoting Rhizobacteria* (PGPR), and are able to stimulate rape and miscanthus growth in the presence of synthetic polymer materials (Janczak et al., 2014a; Janczak et al., 2014b; Da̧browska et al., 2017) or to bind heavy metals (Hrynkiewicz et al., 2015). Similarly, Mahmood et al. found a high potential of *Bacillus* sp. SR-2-1/1 strain belonging to PGPR for remediation of soil containing contaminations generated during the production of textiles (Mahmood et al., 2017). The presence of bacteria in remediation of contaminated soil leads to increased plant production and improvement in the soil condition. The studies showed that inoculation of the natural system with PGPR is one of the practices of sustainable agriculture that helps, for example, to restore soil fertility and decompose soil toxins (Han & Lee, 2005; Saravanan, Madhaiyan & Thangaraju, 2007; Singh, Pandey & Singh, 2011).

Phytoremediation is a biological method of purifying and detoxifying the environment (Mierek-Adamska, Da̧browska & Goc, 2009). It uses the ability of plants to concentrate elements and harmful compounds and transform them into non-toxic compounds or store them in their organs (Karpenko et al., 2015; Kumar et al., 2017; Drewnowska et al., 2020).

In this study, similarly as in previous ones, *Miscanthus x giganteus*, was used, a C-4-type plant from Asia. This plant is characterised by its low requirements with regard to soil condition and fertilisers, its low sensitivity to contaminants, and its high efficiency of biomass production. Miscanthus root exudates are a mixture of water-soluble sugars, amino acids, and organic acids, which may participate in biostimulation of soil bacteria. It is believed that use of prairie grasses for bioremediation is beneficial because these plants have one of the most extended root systems (Christian, Riche & Yates, 2008).

The aim of the conducted studies was to verify whether *B. subtilis* and *B. cereus* strains from the anthropogenically degraded areas, with high metabolic activity, able to grow in the presence of polymer materials can accelerate biodegradation processes of PLA and PET deposited in the soil, besides supporting growth of plants in a polluted environment. The study also examined whether the presence of *M. giganteus* in this system may influence biodegradation of plastics. The obtained results may be used in future as a basis for development of an effective method of bioremediation of areas where synthetic polymer materials are deposited by planting plants designated for biomass production instead of consumption, while maintaining economic and aesthetic aspects.

## Materials and Methods

### Synthetic polymer materials

Polymer samples were made from two types of polymer granulate: PLA (Ingeo^*TM*^ Biopolymer 2003D, Nature Works LLC, USA) and PET (SKYPET –BL 8050, Korea). Films were extruded from the granulates using a laboratory apparatus consisting of a single-screw Plasti-Corder PLV 151 extruder (Brabender, Germany) with a slit die of working width 170 mm and slit height 0.35 mm, and a water-cooled, three-roller smoothing system of roller diameter 110 mm. A screw of length 25D was used with a mixer head of length 8D and compression level of 3:1. The extruder station was additionally equipped with an apparatus necessary to measure the temperature of the heating zones in the plasticising system and the die. PLA and PET films of thickness 0.087 mm were produced (Janczak et al., 2018).

### Microorganisms and bacterial inoculum

In the study, bacteria strains belonging to PGPR were used, *Bacillus* sp. (ML1-2), whose gene sequence available in GeneBank NCBI was verified using BLAST software. It was established that this sequence is 100% homologous to sequences of *B. subtilis*. The *B. subtilis* strain (KM411502.1) was isolated from the mycorrhizosphere of an ectomycorrhizal fungus from degraded soil contaminated with heavy metals. *B. cereus* (HM989919) was isolated from a fruit body of an ectomycorrhizal fungus *Hebeloma mesophaeum* associated with *Salix caprea* from degraded soil contaminated with heavy metals (Hrynkiewicz et al., 2012). Bacteria strains used in the experiments were grown on a solid medium R2A (Oxoid) at 24^∘^C for 2 days. Bacterial inoculum containing 5 ×10^4^cfu/mL was prepared in saline solution (0.9% NaCl pH 7.0). The suspensions prepared in this way were used for further analyzes described in individual sections of this manuscript.

According to some researchers, bacteria *Bacillus* sp. should be used for the biodegradation of polymeric materials, including PLA and PET, and as PGPR bacteria (Bubpachat, Sombatsompop & Prapagdee, 2018; Janczak et al., 2018; Janczak et al., 2020).

### Biochemical characteristic of *Bacillus* strains

Pure cultures of the tested bacterial isolates were checked for their plant growth-promoting properties and for promoting biodegradation of polymeric materials. The following biochemical features were analyzed: production of cellulase on CMC (carboxymethyl-cellulose sodium salt medium) (Hankin & Anagnostakis, 1977), phosphate solubility on PVK substrate (Pikov-skaya’s medium) (Sharma, Kumar & Tripathi, 2011) production of siderophores on CAS (chrome azurol S medium), production of pyoveridin, a fluorescent siderophore in bacteria of the genus *Pseudomonas* (SM) (succinate medium), lipase production (LP), production of protease (SMA) (skim milk agar) (Ghodsalavi et al., 2013).

All media were prepared according to the literature recommendations, then autoclaved at 121 °C for 20 min, cooled and poured into petri dishes to cool and solidify. After inoculation with bacteria, each medium was incubated at 27 °C for 48 h, except for PVK medium which was incubated for 7 days. After incubation, the test results for each tested bacterial isolate were recorded on a 5-point scale: 0–4, where the numbers: 0, 1, 2, 3, 4 - denote in turn: no reaction, low, medium, high and bar- very high intensity of the observed biochemical reaction. The results are presented in Table 1 (Krawczyk et al., 2016).

**Table 1:** Biochemical characteristic of isolated and reference strains.

**Biochemical characteristic/medium**	**Strain**	
****	***B. subtilis***	***B. cereus***
CMK	3	2
PVK	1	1
CAS	1	0
SM	0	0
LP	1	2
SMA	1	1

### The biofilm-forming ability of bacteria

Bacteria isolates of *B. subtilis*, *B. cereus* were pre-incubated in flasks containing nutrient broth (50 mL) for 24 h at 25 °C. Next, optical density (value 1) of each culture was determined in relation to OD_600nm_. The bacterial suspension (0.1 mL) was transferred to a nutrient broth (10 mL) and put onto the PLA and PET surface. The fragments of films (5 ×5 cm) were incubated for 48 h at 25 °C and then dried at room temperature for 40 min. Next, the films were covered with 1% solution of crystal violet which adhered to the biofilm. After 45 min they were washed with distilled water to remove excess dye and dried for 30 min. Subsequently, they were placed in a beaker and 96% ethanol was added to wash away the absorbed dye. The absorbance of alcohol extract was measured using a BioRad spectrophotometer at a wavelength 595 nm. Biofilm abundance was analyzed as absorbance at 595 nm, and performed in triplicate (Adam & Duncan, 2001; Walczak, Richert & Burkowska-But, 2014; Świontek Brzezinska et al., 2018; Świontek Brzezinska et al., 2020).

In order to evaluate the viability of biofilm forming bacteria, microorganisms retained on the filter surface were subjected to viability staining, using a diagnostic LIVE/DEAD set (Invitrogen). Fragments of the film (1 cm^2^ from each film) covered with the aqueous solutions of dyes of LIVE/DEAD BacLight^(TM)^ Bacterial Viability Kit were incubated for 15 min in the dark at room temperature. After the excess dye was removed, the samples were placed on microscope slides and viewed under oil immersion at ×1000 magnification using ECLIPSE 50i fluorescence microscope (Nikon, Japan) (Kumar & Maiti, 2016; Kumar et al., 2017; Richert & Dąbrowska, 2021).

### Plant material and soil parameters

Plant material consisted of giant Miscanthus (*M. giganteus*) seedlings from a two-year cultivation in a field and 7-weeks-old plants from the *in vitro* cultures. Seedlings, containing 4–5 shoots, were obtained by mechanically dividing rootstock.

In the pot experiment, two-year old compost soil was used, prepared by mixing plant fragments, sand, clay and peat at equal ratios. The compost soil contained: 8.02% MO, 3.71% C_org_, 0.30% Nt, 12.0 C/N, 1.29% CaCO_3_, pH_H2O_7.5, pH_KCl_7.1.

### Pot experiments

Plants of *Miscanthus* were obtained from *in vitro* cultures. Pot experiment was conducted in three variants of inoculation: control (non-inoculated) plants and inoculated with *B. cereus* and *B. subtilis* strain in growth substrate (sterile mixture of sand and vermiculite, 1:1). Plants (*n* = 30, for each variant) were cultivated in the pot (a volume of each pot 0.3 l) in the temperature: 24 °C ± 2 °C and light/dark: 16/8) for 10 weeks and irrigated with Hoagland medium. The growth parameters such as: fresh and dry biomass of shoots and roots, number of leaves, length of shoots and roots were assessed after 10 weeks.

Studies on the biodegradation of PLA and PET in soil were carried out for 6 months (March–August) under greenhouse conditions in 2200-cm^3^ pots. In the first week of growing the soil was inoculated with one mL of bacterial suspension.

Pieces of PLA and PET film were placed in the soil. Their dimensions were: 30 ×30 mm, 100 ×15 mm and 130 ×110 mm (3 pieces of each), thickness of film had 0.087 mm. The use of various film sizes was related to their subsequent use in various methods for testing changes in biodegradation on plastics. The following experiment systems were used: soil + film (variants with PLA or PET), soil + film + bacteria strain (variants: PLA + *B. subtilis*, PLA + *B. cereus*, PET + *B. subtilis*, PET + *B. cereus*), soil + plant + film (PLA + *M. giganteus*, PET + M*. giganteus*), soil + plant + film + bacteria strain (PLA + *M. giganteus* + *B. subtilis*, PLA + *M. giganteus* + *B. cereus*, PET + *M. giganteus* + *B. subtilis*, PET + *M. giganteus* + *B. cereus*). Three biological replicates were produced for each variant. After 6 months, the film samples were removed from the soil and washed with 70% EtOH and then three times with water. The films were dried on filter paper for 12 h at RT (ISO 846, 1997) used to demonstrate biodegradation changes.

### Assessment of film biodegradation

#### Visual and microscopic assessment of PLA and PET

A microscopic analysis of structural changes of the film surface was carried out using an SEM (Hitachi SU 8010, Japan). Fragments were cut from the 30 ×30 mm samples, and then sprayed with gold to a thickness of 1 nm using a gold dust sprayer (Cressington Sputter Coater 108 auto, UK) with a dust-layer thickness meter (Cressington Thickness Monitor mtm10, UK). Pictures were taken at 1000 × magnification. Energy Dispersive X-ray analysis (EDX) was performed using the SEM-EDX module (Thermo Scientific Ultra Dry, USA). The EDX spectrum of the sample surface allowed a semi-quantitative analysis of the elementary composition to the depth of ca. 1 μm (Varesano, Dall’acqua & Tonin, 2005). During the EDX analysis, a voltage of 20 kV and current of 15 μA was applied to the prepared samples. The analysis of the elemental composition of the surface lasted 30 s at ×100 magnification and a working distance of 15 mm. The experiment was repeated in 5 replicates for each variant containing either PLA or PET film.

#### Loss of film weight

The analysis was conducted in accordance with the standard ISO 846 (1997). The film was weighed on an analytical balance to the nearest 0.001 g before incubation in the soil, and after being removed from the soil and thoroughly cleaned. The loss of weight expressed as a percentage (%) was the difference in weight of the film before and after incubation in the soil.

#### Film tensile strength test

The tensile strength of polymers was analysed on the 100 ×15-mm films using TIRATest 27025 equipment (TIRA GmbH, Germany). The following values were determined: total elongation at break –AB (%) and breaking strength –FB (N). The test was conducted in accordance with the standard ISO 527 (2012).

#### Fourier transform infrared spectroscopy (FTIR)

Infrared spectroscopy (FTIR) was applied using the Attenuated Total Reflectance (ATR). FTIR spectra were recorded in the 400–4000 cm^−1^ range using an FTIR Cary 630 spectrometer (Agilent, USA) equipped with a diamond crystal (spectral resolution <2 cm^−1^). Degradation processes may cause the detachment of substituents (giving signals in the IR spectrum) or the breaking of C-C and C-H bonds in the main chain with the simultaneous formation of e.g., carbonyl groups. If the polymer contains functional groups characterized by absorption in a certain range, the degree of polymer degradation can be determined by recording the absorption spectra and analyzing the changes in individual bands. To compare the extent of biodegradation of the PLA and PET polymers, the carbonyl index (%) was used to determine the ratio of band intensity from the carbonyl group to the band intensity of the group that was not changed during biodegradation. An elevated carbonyl index relative to the control samples indicates that biodegradation changes are taking place due to the onset of oxidation of the sample.

#### Film permeability to H_2_O↑, O_2_ and CO_2_ gases

130 ×110-mm PLA and PET film samples were used for analysis of changes in gas permeability to water vapor (H_2_O↑) according to standard using a Water Vapor Permeability Tester (Lyssy L100-5000, Switzerland), and to oxygen (O_2_) and carbon dioxide (CO_2_) according to standard ASTM E-398-03 (2009) and ASTM D-1434 (1434) using a Manometric Gas Permeability Tester (Lyssy L80-5000, Switzerland).

## Results and Discussion

### Biochemical characteristics

There are many biochemical features of bacteria that have a beneficial effect on plant growth and accelerate the biodegradation processes of polymeric materials (Robertson et al., 2018; Bandopadhyay, Sintim & DeBruyn, 2020). The study investigated five selected biochemical features, the relationship of which with the increase in shoot length and the increase in the amount of root extracts has been scientifically proven (Ghodsalavi et al., 2013). On the basis of the conducted tests, it was determined which of the tested isolates show biochemical properties potentially favorably influencing plant growth and biodegradation. These results are presented in Table 1.

Among the tested isolates, strong intensities of the biochemical reaction on the CMC medium indicating the presence of the cellulase enzyme and enzymes strongly dissolving inorganic phosphates on the PVK medium were noted. In the case of the CAS medium on which the ability to produce siderophores by bacteria was tested, a positive result was recorded for *B. subtilis*. In contrast, in the SM medium, no positive reaction was noted for any of the isolates for the presence of pyoveridine, a fluorescent siderophore produced mainly by *Pseudomonas*. Both *B. subtilis* and *B. cereus* have a strong biochemical response to lipases (LP) and proteases (SMA).

There are metabolic differences between strains of the same species in terms of the examined features, which may indicate the ability of bacteria to adapt to the environment they currently occupy. Studying these differences can contribute to a better understanding of the bacteria’s mechanisms of action and help identify genetic markers encoding bacterial biochemical traits that support plant growth.

### Biofilm in PLA and PET surfaces

**Figure 1:**
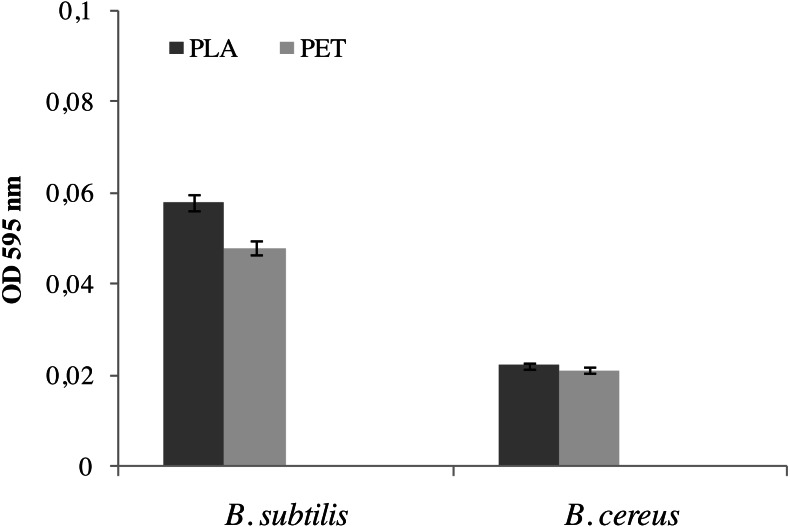
Biofilm formation by bacteria *B. cereus* and *B. subtilis* on PLA and PET films. Values are expressed as mean ± SD (*n* = 3).

The formation of a biofilm by bacteria is the first step in the biodegradation of polymers ( Richert & Da̧browska, 2021; Janczak et al., 2020). Figure 1 shows the results of biofilm formation by *Bacillus* strains. The amount of biofilm formed by the *B. subtilis* bacteria on the individual types of film was significantly greater than in the case of the *B. cereus* strain. A 17% larger biofilm was noted on the surface of the film made of PLA than on the PET film. The biofilm formed by *B. cereus* on PLA and PET was practically the same size at 0.022 OD_595nm_.

**Figure 2:**
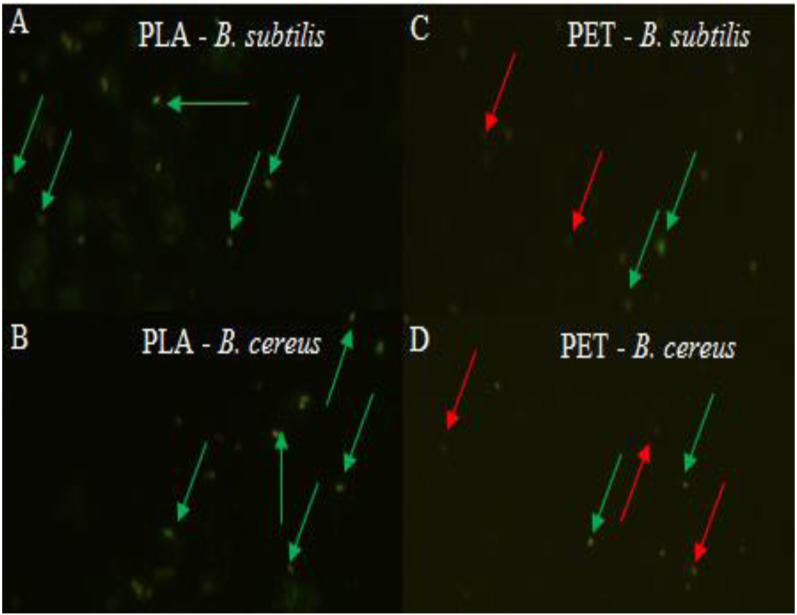
Biofilm formed by (A, C) *B. subtilis* and (B, D) *B. cereus* on the surface of PLA and PET by using the LIVE/DEAD method, live bacteria—green, dead bacteria—red.

At the same time, the bacteria forming the biofilm on the PLA surface had a high viability, and therefore the number of microorganisms was greater than on the PET surface, which was clearly confirmed by the LIVE/DEAD analysis (Fig. 2). There is no doubt that a biofilm has formed on the surface of the polymers degrades these materials. However, the rate of biodegradation depends on activity of microorganisms and type of polymers. Polymers withwith high molecular weight have a slower rate of degradation than those with they have a low molecular weight and take many years to degrade long-chain polymers for simple hydrocarbons (Kim & Park, 2010; Lee, Kim & Song, 2014; Wang et al., 2020).

### The growth of miscanthus in presence of *Bacillus* sp. strains

The effect of *B. cereus* and *B. subtilis* bacterial strains on the growth of miscanthus was tested (Table 2). The plant inoculation with bacteria stimulates the growth of plant roots and shoots. There was also an increase in the biomass of roots and shoots, but the result was not statistically significant. The presence of bacteria increased the survival rate of plants.

**Table 2:** The effect of *B. cereus* (BC) and *B. subtilis* (BS) on the growth of *Miscanthus* for 10-weeks cultures.

**Strain**	Level of survival^*^	**Shoot length [cm]**	**Root length [cm]**	**Living leaves number**	**Fresh wright [g]**		**Dry wright [mg]**	
****	****	****	****	****	**Shoot**	**Root**	**Shoot**	**Root**
control	58.3	37.12 ± 2.07 b	12.61 ± 0.65 b	5.0 ± 0.2 ab	0.88 ± 0.12 bc	0.24 ± 0.04 bc	108.66 ± 15.14 bc	19.00 ± 2.81 bc
BS	91.7	46.19 ± 1.89 a	16.42 ± 0.83 a	4.7 ± 0.3 b	1.11 ± 0.10 ab	0.40 ± 0.05 ab	146.58 ± 15.08 ab	32.33 ± 4.34 ab
BC	69.4	45.2 ± 1.6 ab	11.2 ± 0.7 a	5.7 ± 0.3 ab	1.11 ± 0.09 abc	0.30 ± 0.3 a	147.75 ± 11.57 abc	29.69 ± 2.41 ab

**Notes.**

* Statistically significant differences between samples for each parameter are marked with different letters.

Janczak et al. (2020) have conducted similar research work using other bacterial strains (Janczak et al., 2020). *B. cereus* has been shown increase biomass production of *Trifolium repens* (Azcon et al., 2010) and *Salix viminalis* (Hrynkiewicz et al., 2012).

### Assessment of film surface

**Figure 3:**
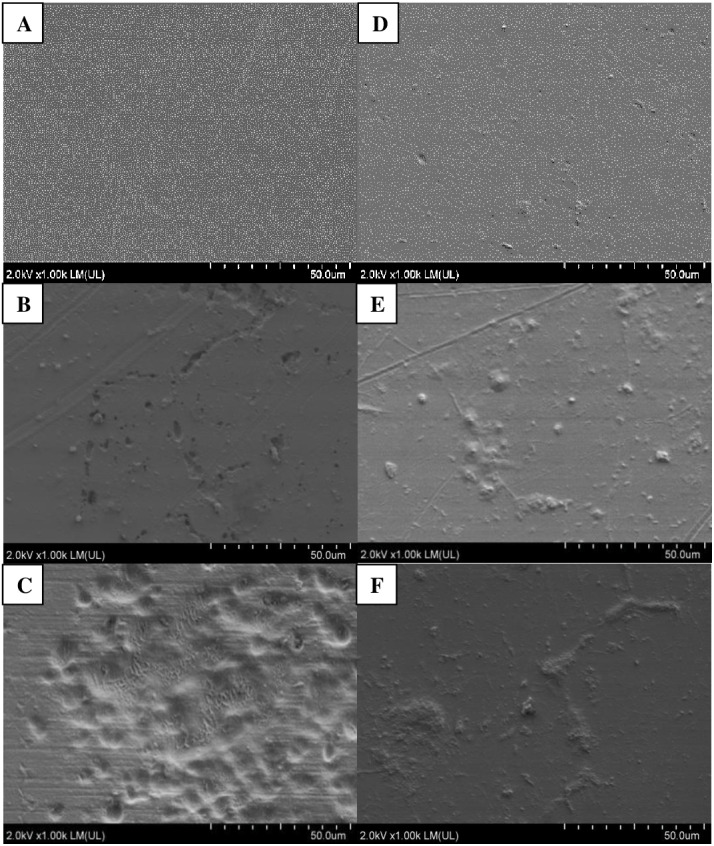
Changes in the surface structure of PLA and PET films following six months of incubation in soil: A–C (PLA); D–F (PET) variants respectively: Control (A, D), *B. subtilis* (B, F), *B. cereus* (C, F) analysed using SEM.

Photographic documentation of film samples was prepared after six months of incubation in soils inoculated with bacteria and in the control, not containing inoculum (Fig. 3). Analyses of both films, PLA and PET, using SEM, showed presence of microorganisms on the surface of films in variants inoculated with bacteria from the *Bacillus* genus. More changes were found on PLA film, versus PET.

**Figure 4:**
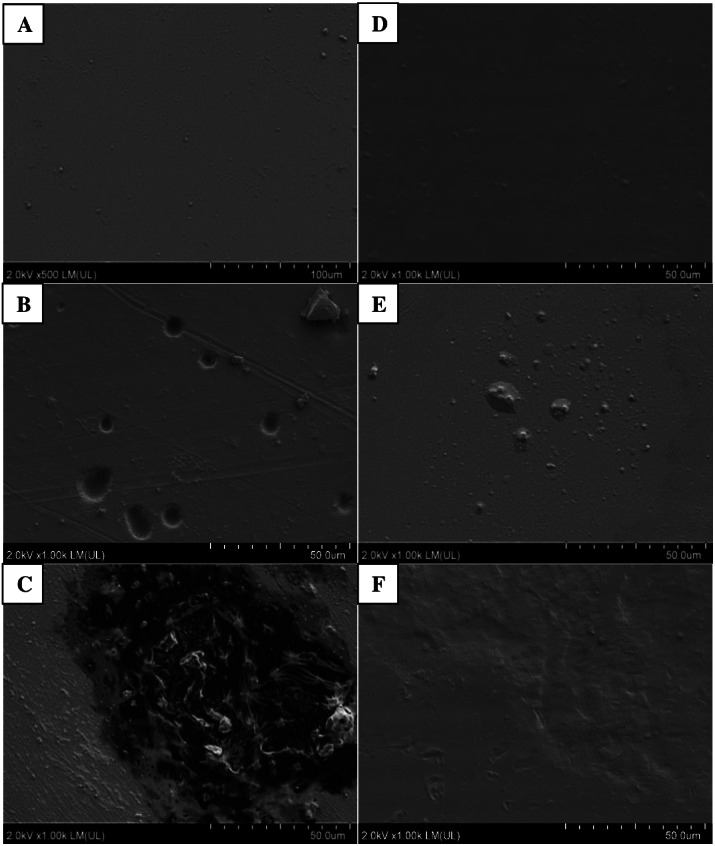
Changes in the surface structure of PLA and PET films following six months of incubation in soil with plants: A–C (PLA); D–F (PET) variants respectively: Control (A, D), *B. subtilis* (B, F), *B. cereus* (C, F) analysed using SEM.

Following incubation in soil, the samples of PLA film were changed; cracks and chips were observed in the film. These changes were more pronounced in variants with the plant. In variants without the plant, only films incubated in soil in the presence of *B. cereus* were cracked, and in SEM images, numerous cavities were additionally seen in the film. These bacteria also caused degradation changes in the PLA surface in variants with the plant, which were visible as worn areas with irregular fragments of non-degraded polymer. The presence of *M. giganteus* contributed to formation of cavities in the PLA film, possibly due to its contact with roots and their exudates (Figs. 3–4).

Following PET film incubation in soil inoculated with bacteria, no film deformation was observed. Similar changes were present in variants with the plant. This may be an effect of root exudates or autochthonous microorganisms (Fig. 3). Similar results have been reported by others (Ziembinska & Gatlik, 2012; Lee, Kim & Song, 2014; Hermanova et al., 2015).

### Loss of film weight

Mechanism and process of biodegradation depends on the molecular weight of the plastic and/or its composition (e.g., addition of plasticizers, dyes, flame retardants), on the presence of microorganisms and on environmental conditions (e.g., soil reaction, temperature) (Bano et al., 2017). Most polymers are too large to pass through cell membranes. The biological degradation of polymers actively involves at least two categories of enzymes: extracellular depolymerases and intracellular depolymerases (Muthukumar & Veerappapillai, 2015). During degradation, exoenzymes from microorganisms break down complex polymers, resulting in smaller short-chain molecules, e.g., oligomers, dimers and monomers, that are small enough to pass through semi-permeable outer bacterial membranes and then be used as a carbon and energy source. The process is called “depolymerization”. The microorganisms use the plastics as a source of carbon when nutrients are in poor supply or inaccessible.

Therefore, there is a general opinion that PLA may contribute to environmental pollution (Karamanlioglu & Robson, 2013). In films from variants with soil inoculated with *Bacillus*, a significant loss of film weight was found for PLA film versus the control (film from non-inoculated soil), excluding PLA from soil inoculated with *B. subtilis*. A ca. 70% loss of weight was demonstrated versus the control film for all the remaining variants inoculated with bacteria. Presence of the plant resulted in ca. 55% loss of weight (Table 3).

**Table 3:** Mass reduction versus control of PLA film in inoculated and non-inoculated soil.

**Sample name**		**mass loss [%]**	**Legend**
soil	*C*^a^	1.13 ±0.03^a^^*^	^a^ Control –film in non-inoculated soil.
	BS^b^	1.20 ±0.03^a^	^b^ Variant in soil inoculated with *B. subtilis*.
	BC^c^	3.42 ± 0.06^d^	^c^ Variant in soil inoculated with *B. cereus*.
soil+plant	MG^d^	2.47 ± 0.05^b^	^d^ Variant with a plant, *M. giganteus*, in non-inoculated soil.
	MG+BS^e^	3.25 ± 0.07^c^	^e^ Variant with a plant, *M. giganteus*, in soil inoculated with *B. subtilis*.
	MG+BC^f^	4.62 ± 0.05^e^	^f^ Variant with a plant, *M. giganteus*, in soil inoculated with *B. cereus*.

**Notes.**

* Statistically significant differences between samples for each parameter are marked with different letters.

### Analyses of film properties (EDX, FTIR, Film tensile strength test)

To demonstrate biodegradation changes in PLA and PET films, oxygen and carbon content (%_*w*_) on the surface of analysed samples was verified using the EDX analysis. The oxygen-to-carbon ratio O/C (%) was calculated, and in all samples of PLA film it was higher than in the control film (Table 4), and this indicates biodegradation changes (Vieira, Guedes & Tita, 2013; Lv et al., 2017).

**Table 4:** Results of an analysis of film properties for PLA and PET after six months of incubation in control soil and in soil inoculated with bacteria. (*Key as in Table 3).

** Sample name**		**O/C [%]**	**Carbonyl index [%]**	**AB [%]**	**FB [N]**
PLA + soil	C	154.4 ±0.95^a^	802.6 ±23.20^b^	3.68 ±0.10^b^	82.7 ±1.18^b^
	BS	154.0 ±0.07^a^	695.2 ±3.43^a^	3.36 ±0.16^b^	74.8 ±3.32^ab^
	BC	159.5 ±0.54^c^	715.4 ±5.82^a^	2.39 ±0.20^a^	76.3 ±1.08^ab^
PLA + soil+plant	MG	153.3 ±0.20^a^	713.8 ±12.41^a^	2.72 ±0.07^a^	81.4 ±6.06^b^
	MG+BS	157.1 ±0.17^b^	683.7 ±21.22^a^	2.49 ±0.10^a^	70.4 ±2.15^ab^
	MG+BC	160.6 ±0.41^c^	710.1 ±7.85^a^	2.34 ±0.21^a^	64.3 ±3.27^a^
PET + soil	C	96.8 ±0.10^a^	115.1 ±0.93^b^	5.22 ±0.16^d^	88.6 ±1.28^ab^
	BS	99.3 ±0.30^b^	113.6 ±2.48^b^	5.20 ±0.04^d^	89.2 ±0.70^ab^
	BC	98.0 ±0.18^b^	99.1 ±0.41^a^	4.32 ±0.06^b^	92.6 ±1.27^ab^
PET + soil+plant	MG	98.6 ±0.02^a^	122.1 ±0.20^c^	4.75 ±0.17^c^	90.9 ±2.54^ab^
	MG+BS	97.6 ±0.14^b^	118.2 ±0.16^b^	3.78 ±0.14^a^	88.0 ±1.74^ab^
	MG+BC	101.5 ±0.14^c^	102.9 ±2.17^a^	3.79 ±0.20^a^	87.7 ±2.15^a^

**Notes.**

* Statistically significant differences between samples for each parameter are marked with different letters.

The most significant increase in this value was observed for samples inoculated with *B. cereus* in the presence of the plants, for PLA and PET a like. For the PLA film, an increase in the O/C ratio was additionally observed in variants inoculated with one of the *Bacillus* strains in the presence of the plants, and with *B. subtilis* strain in soil without plants (Table 4). For the PET film, a slight increase in the O/C ratio was noticed following inoculation with each of the bacteria strains (Table 4). In our six-month study (180 days), changes of 0.3 and 0.6 in the O/C ratio were noted in the presence of *B. subtilis* and *B. cereus*, respectively.

In the ATR spectrum for the PLA film obtained in FTIR analysis the following bands were identified: 1746 cm^−1^ –absorption of vibrations of the C=O group; 1450 cm^−1^ –absorption of asymmetric vibrations of the CH_3_ group; 1265 cm^−1^, 1179 cm^1^, 1126 cm^−1^, 1078 cm^−1^, 1041 cm^−1^ –absorption of stretching vibrations of O-C-C; 866 cm^−1^ –absorption of vibrations of the O-CH_3_-CH_3_ group; 753 cm^−1^, 701 cm^−1^ –absorption of deformation vibrations of αCH_3_ (Cai, Lv & Feng, 2013). The area at the wave number of 1746 cm^−1^ was more closely analysed, as it is characteristic for the carbonyl group, which is susceptible to biodegradation processes. The carbonyl index was calculated in relation to absorption from asymmetric vibrations of the CH_3_ group at the wave number of 1450 cm^−1^. A drop in the value of the carbonyl index was observed in all the remaining variants versus the control, at a comparable level.

In the ATR spectrum for the PET film the following bands were identified: 2960 cm^−1^ –absorption of asymmetric, stretching vibrations of the C-H group; 1712 cm^−1^ –absorption of vibrations of the C=O group; 1450 cm^−1^ –absorption of vibrations of the C-H group; 1407 cm^−^^1^ –absorption of deformation vibrations of the C-H group; 1235 cm^−1^ –absorption of asymmetric, stretching vibrations in the C-C-O group with a carbon atom in an aromatic ring; 1087 cm^−1^ –absorption of asymmetric, stretching vibrations of the C-C-O group; 871 cm^−1^ –absorption of vibrations of the C-H group in an out-of-plane aromatic ring; 722 cm^−1^ corresponding to absorption of rotational vibrations of the C-H group in the aromatic ring (Cai, Lv & Feng, 2013). The area at the wave number of 1712 cm^−1^ was more closely analysed, as it is characteristic for the C-H group in the aromatic ring at the wave number of 871 cm^−1^. The most significant decrease in the value, indicating biodegradation changes, was observed for the PET film from soil inoculated with *B. cereus* (14% versus the film from non-inoculated soil) and containing plants. However, the highest value was noted for the film in non-inoculated soil with the plant.

The tensile strength analysis of the film based on measurements of elongation at break –AB (%) and the break force –FB (N) demonstrated biodegradation changes in both films. Changes in strength were visible as a reduction in the break force (FB) in the PLA film, and as shortening of elongation at break (AB) for PET (Table 4). For PLA, lower AB was noted for all analysed variants, excluding the film incubated in soil with *B. subtilis* without plants. A greater reduction in strength was observed for FB values, where the most significant result was obtained in the variant inoculated with *B. cereus* with the plant (Table 4). For PET, the most significant reduction in AB was noted for variants with plants in soil inoculated with one of the strains, and in the variant without the plant in soil inoculated with *B. cereus*. The presence of the plant had a stronger effect on loss in PET tensile strength than inoculation with *B. subtilis* (Table 4). Similar results have been reported by others (Kim & Park, 2010; Iwanczuk et al., 2013).

### Analysis of film permeability to H_2_O↑, O_2_ and CO_2_ gases

A parameter frequently analysed as an indicator for plastic degradation is film permeability to gases such as water vapour, oxygen or carbon dioxide (Hulsmann & Wallner, 2017). Film permeability to H_2_O↑, O_2_ and CO_2_ gases was measured for the PET film only. The PLA film was too defragmented to prepare samples of 130 ×110 mm required for this analysis. During the biodegradation process permeability to gases increases. An elevated permeability to H_2_O↑ (g/m^2^ day) was noted only for variants inoculated with bacteria in presence of the plant. On the other hand, the greatest increase in permeability to O_2_ (g/m^2^ day) was noted after inoculation with *B. cereus*, regardless of whether the plants were present or not. A significant effect of the plants was also noted, both for the non-inoculated variant and in the presence of *B. subtilis*. An increase in permeability to CO_2_ (mL/m^2^day) was noted after inoculation with *B. cereus*, regardless of whether the plants were present or not, and following soil inoculation with *B. subtilis* without plants. In our studies, an increase in permeability to water vapor by ca. 1 g/m^2^day was demonstrated for samples from soil inoculated with *Bacillus* strains. An increase in permeability to oxygen and carbon dioxide of 15 mL/m^2^ day and 17 mL/m^2^ day, respectively, was also found for film incubated in the presence of *B. cereus*, and by ca. 6% and 9%, respectively, in the presence of *B. subtilis*. This data implies that the strains used contribute to degradation changes in PET film during its incubation in soil (Table 5).

**Table 5:** Permeability to gases (H_2_O↑, O_2_, CO_2_) of PET films following six months of incubation in soil. (*Key as in Table 3).

**Sample name**		**H_2_O ↑ [g/m^2^ day]**	**O_2_ [mL/m^2^ day]**	**CO_2_ [mL/m^2^ day]**
soil	C	13.98 ±0.02^a^	49.54 ±0.01^a^	145.95 ±0.88^a^
	BS	13.88 ±0.02^a^	49.88 ±0.31^a^	155.12 ±4.23^b^
	BC	13.97 ±0.01^a^	63.04 ±0.08^c^	162.61 ±4.37^b^
soil+plant	MG	13.95 ±0.01^a^	52.87 ±0.74^b^	145.37 ±2.18^a^
	MG*+* BS	14.80 ±0.18^b^	54.76 ±0.47^b^	149.17 ±0.46^a^
	MG*+* BC	14.94 ±0.14^b^	64.66 ±1.94^c^	162.92 ±3.88^b^

**Notes.**

* Statistically significant differences between samples for each parameter are marked with different letters.

## Conclusions

Many previous studies focused only on identifying and studying the role of microorganisms participating in biodegradation of plastics forming a significant part of waste deposited at municipal landfills. Endospores produced by microorganisms of the genus *Bacillus* are very resistant to unfavorable environmental conditions (heat, drying), as well as to many disinfectants.

Our studies show that analyzed bacterial strains of *B. subtilis* and *B. cereus*, originating from anthropogenic degraded areas, show high metabolic activity, are able to grow in the presence of PLA and PET, and can accelerate the degradation of these polymeric materials.

Our studies show that one of the methods for soil remediation may be the use of *Bacillus* strains combined with planting of Miscanthus, a plant of economic importance. We suggest that this system may represent a real strategy for transforming industrially contaminated soil into an environmentally friendly area which is biologically and economically productive.

##  Supplemental Information

10.7717/peerj.10957/supp-1 Supplemental Information 1 Raw Data

## Additional Information and Declarations

### Data Availability

The following information was supplied regarding data availability: Raw data are available as a Supplemental File.
